# Efficacy and safety of tolvaptan for refractory fluid collection and edema in the terminal cancer patients

**DOI:** 10.20407/fmj.2021-005

**Published:** 2022-05-25

**Authors:** Norimasa Tsuzuki, Masanobu Usui, Akihiro Itoh, Akihiko Futamura, Kazuki Imai

**Affiliations:** 1 Department of Surgery and Palliative Medicine, Fujita Health University, School of Medicine, Toyoake, Aichi, Japan; 2 Department of Pharmacy, Fujita Health University Nanakuri Memorial Hospital, Tsu, Mie, Japan

**Keywords:** Tolvaptan, Palliative medicine, Terminal cancer, Edema

## Abstract

**Objective:**

Tolvaptan, a vasopressin V_2_ receptor antagonist, is an oral diuretic. Patients with terminal cancer develop marked fluid retention, and oral diuretics other than tolvaptan have been used as treatments without clear therapeutic effects. Herein, we aimed to study the efficacy and safety of tolvaptan in patients with terminal cancer.

**Methods:**

Tolvaptan was administered at a dose of 7.5 mg/day to 29 patients (median, 72 years) between August 2017 and February 2020. The duration of tolvaptan treatment ranged from 1 to 85 days (mean, 18.5 days).

**Results:**

Median albumin (Alb) and transthyretin (TTR) levels on admission were 2.3 g/dL (1.2–4.2 g/dL) and 8.9 mg/dL (2.1–38.2 g/dL), respectively. Median Alb and TTR levels 1 month after treatment initiation remained at 2.3 g/dL (0.8–2.9 g/dL) and 8.6 mg/dL (0.8–23.7 mg/dL), respectively. Regarding renal function indicators, median blood urea nitrogen (BUN) and creatinine levels on admission were 19.9 mg/dL (8.6–49.3 mg/dL) and 0.81 mg/dL (0.38–2.25 mg/dL), respectively. Median BUN and creatinine levels 1 month after treatment initiation were 23.4 mg/dL (13.5–34.0 mg/dL) and 0.91 mg/dL (0.39–2.41 mg/dL), respectively. No patients had hypernatremia on admission, and no effects of tolvaptan on the blood sodium level were found 1 month after treatment initiation. The median potassium level on admission was 4.2 mEq/dL (2.9–5.0 mEq/dL); tolvaptan treatment had no effects on blood potassium level.

**Conclusions:**

Tolvaptan is effective and safe for treating fluid retention refractory to conventional diuretics in patients with terminal cancer.

## Introduction

Tolvaptan, a vasopressin V_2_ receptor antagonist developed in Japan, is an oral diuretic that causes excess fluid retained in the body to be excreted in urine. Although hospitalization is required to initiate this treatment, data from the clinical use of tolvaptan for heart failure and hepatic cirrhosis have demonstrated the efficacy and safety of tolvaptan.^1–3^ Significant fluid retention is also observed in patients with terminal cancer. Although oral diuretics other than tolvaptan have been used for their treatment, clear therapeutic effects could not be obtained in some cases. In these patients, fluid retention is often refractory to drug treatment. Moreover, nutritional intake is insufficient in such patients because of the restrictions on food intake and volume of infusion, which are required to prevent edema from worsening. Furthermore, when marked edema persists, ascites and pleural effusion can cause abdominal distension and dyspnea, resulting in reduced activities of daily living and prolonged hospitalization. Tolvaptan, which is also used to treat patients with heart failure, has been reported to be effective in the treatment of ascites associated with hepatic cirrhosis; however, it is not commonly used in the field of palliative medicine. At our hospital, we have been using tolvaptan proactively for patients with terminal cancer with refractory ascites and hypoalbuminemia associated with hepatic impairment due to hepatic metastasis or other causes because such patients can be diagnosed with a type of hepatic cirrhosis at least.^4^

In this study, we investigated the efficacy and safety of tolvaptan for the treatment of refractory marked fluid retention in patients with terminal cancer.

## Methods

This study included 29 patients with terminal cancer who developed marked fluid retention between August 2017 and February 2020 and who did not respond to other diuretics. These patients were administered tolvaptan at a dose of 7.5 mg/day. In 7 of these patients, tolvaptan was prescribed by a primary care physician and the treatment was continued after admission to our institution, whereas in 22 patients, the treatment was newly initiated upon admission to our hospital. Diuretics used before the initiation of tolvaptan administration were as follows: loop diuretics in 19 patients, K-sparing diuretics in 11, thiazide diuretics in 1, none in 3, and two oral agents in 5. Among these patients, 13 were men and 16 were women (aged 46–93 years; median, 72 years). The sites of the primary lesions were as follows: gastrointestinal in 21 patients (pancreas, bile duct, liver, esophagus, stomach, colon, and rectum in 5, 4, 3, 1, 3, 3, and 2 patients, respectively), lungs in 2, gynecological in 2, urological in 2, and other in 2 (Table 1). Patients underwent the following assessments at least once a month: body weight, serum albumin level, and serum transthyretin level as parameters in nutritional assessments, and blood urea nitrogen (BUN), creatinine, and electrolytes as parameters in renal function assessments.

The research protocol was evaluated and approved by the ethics committees of Fujita Health University School of Medicine (registry no. HM20-105: study of prognosis by the ratio of CRP/TTR, TTR, and CRP in terminal cancer patients) and of each facility. All data were analyzed using the Software Package for Social Sciences (SPSS) for Macintosh version 24.0 (IBM Company, Chicago, IL, USA). The data regarding the continuous variables were expressed as mean values±SD, and statistical significance was determined using Student’s t-test or Mann–Whitney U test.

## Results

The duration of tolvaptan administration ranged from 1 to 85 days (mean, 18.5 days). Performance status (PS) scores at the initiation of tolvaptan treatment were as follows: PS1 in 2 patients, PS2 in 9, PS3 in 10, and PS4 in 8; most patients had decreased PS scores and those with a PS score of ≥3 accounted for 62.1% (18 patients). All patients, however, were capable of oral intake. Fluid retention conditions were as follows: trunk edema as well as pleural fluid and ascites retention in 12 patients, lower leg edema in 8, ascites retention in 5, pleural effusion in 2, pleural fluid and ascites retention in 1, and anasarca in 1. The median duration of survival was 38 days (range, 9–345 days) (Table 1).

**Figure 1:**
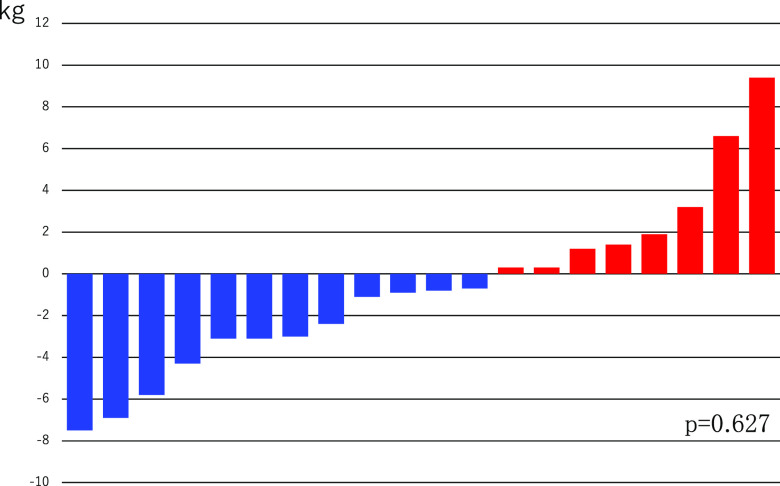
Changes in the body weight before and one month after administration of tolvaptan.

The median body weight upon admission was 56.9 kg (range, 34.9–106.3). PS scores at 1 month after treatment initiation were evaluated in 14 patients: PS1, 1 patient; PS2, 5; PS3, 4; and PS4, 4. PS3 and PS4 were observed in all 8 patients (57.1%), which did not differ in number. Regarding the change in PS scores, three patients showed improvements, eight showed no changes, and three showed deterioration. Thus, the PS scores were maintained or improved for 11 patients (78.6%), even in those with terminal cancer. The median body weight in 20 patients whose body weight changes could be monitored for at least 1 month was 57.6 kg (range, 35.2–99.4 kg), and >50% these patients (12 patients; 60.0%) showed a decrease in body weight and 8 (40.0%) showed an increase in weight. In the 12 patients who showed a decrease in body weight, the median weight decrease was 3.1 kg/month (range, 0.8–7.5 kg/month), and the decrease was steady. In the eight patients who showed an increase in their body weight, the median weight increase was 1.7 kg/month (range, 0.3–9.4 kg/month). Except for two patients who had refractory cachexia and whose body weight increases were 6.6 kg and 9.4 kg, the increases in the remaining five patients were <2 kg, showing mild changes compared with those in patients showing a decrease in their body weight (Figure 1). Six of the eight patients with lower leg edema showed improvement.

**Figure 2:**
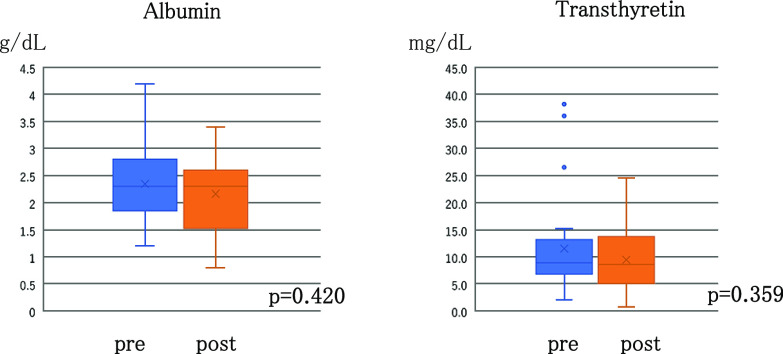
Changes in the albumin and transthyretin before and one month after administration of tolvaptan.

Regarding nutritional status, the albumin levels of all patients were measured at admission; the median albumin level was 2.3 g/dL (range, 1.2–4.2 g/dL), which was markedly low. The transthyretin level at admission was measured in 28 patients; the median transthyretin level was 8.9 mg/dL (range, 2.1–38.2 mg/dL), which was also abnormally low (Table 2). The median albumin level 1 month after treatment initiation in 24 patients for whom the data were available was 2.3 g/dL (range, 0.8–2.9 g/dL), which did not differ from the level measured at admission; the albumin levels in patients who survived for at least 1 month decreased slightly, but the change was not significant (p=0.420, Figure 2). The median transthyretin level 1 month after treatment initiation in 21 patients for whom the data were available was 8.6 mg/dL (range, 0.8–23.7 mg/dL); the decrease from the level at admission was 0.3 mg/dL. Except for one patient showing a decrease of 21.8 mg/dL, the transthyretin levels in patients who survived for at least 1 month remained virtually unchanged with five patients showing an improvement (p=0.359, Figure 2). The values 2 and 3 months after treatment initiation could be measured only in a few patients but showed no statistically significant results.

**Figure 3:**
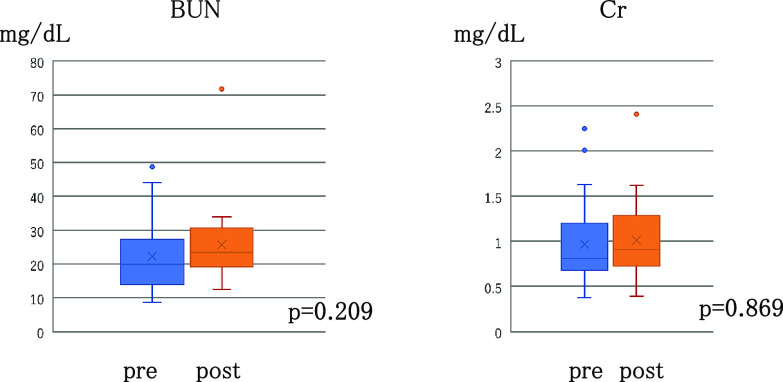
Changes in the BUN and Cr(creatinine) before and one month after administration of tolvaptan.

Regarding renal function, BUN and creatinine levels at admission were measured in all patients. The median BUN level was 19.9 mg/dL (range, 8.6–49.3 mg/dL), and 12 (41.4%) patients had BUN levels of ≥20 mg/dL at admission. The median creatinine level was 0.81 mg/dL (range, 0.38–2.25 mg/dL), and nine (31.0%) patients had creatinine levels of ≥1.0 g/dL at admission. These measurements indicated that >30% of the patients already had renal dysfunction when they were admitted (Table 2). In 22 patients whose BUN levels could be measured 1 month after treatment initiation, the median level was 23.4 mg/dL (range, 13.5–34.0 mg/dL), showing only a slight increase compared with the level measured at admission (p=0.209, Figure 3). Creatinine levels could be measured in 21 patients 1 month after the treatment initiation, and the median was 0.91 mg/dL (range, 0.39–2.41 mg/dL), showing virtually no changes from the level measured at admission. In particular, no patients with normal creatinine levels at admission (<1.0) showed an increase to >1.0 (p=0.869, Figure 3). Similarly, BUN/creatinine levels 2 and 3 months after treatment initiation could be measured only in a few patients but revealed no statistically significant results.

**Figure 4:**
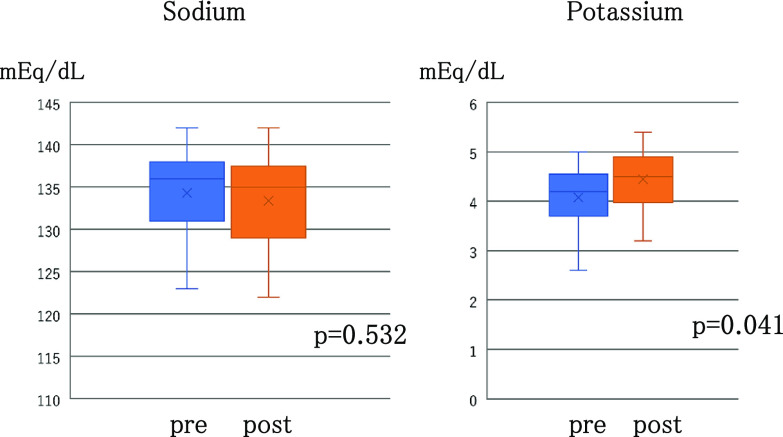
Changes in the serum sodium and potassium before and one month after administration of tolvaptan.

Regarding electrolytes, the median level of serum sodium at admission was 136 mEq/dL (range, 123–142 mEq/dL), and no patients had hypernatremia when they were admitted (Table 2). The median level of serum sodium 1 month after treatment initiation was 135 mEq/dL (range, 124–142 mEq/dL), showing no effects of tolvaptan (p=0.532, Figure 4). The median potassium level on admission was 4.2 mEq/dL (range, 2.9–5.0 mEq/dL), and no patients had severe hyperkalemia (Table 2). The median potassium level 1 month after treatment initiation was 4.5 mEq/dL (range, 3.2–4.5 mEq/dL); the change from the level on admission was normalization rather than elevation through the use of high-calorie infusion and correction of low potassium level; this change was not appreciably associated with the effects of tolvaptan. After treatment initiation, no patients required dialysis or experienced adverse reactions such as arrhythmia (p=0.041, Figure 4).

## Discussion

The recent advent of tolvaptan has altered the recommended regimens for diuretic treatment of ascites in patients with hepatic cirrhosis. Tolvaptan selectively antagonizes the vasopressin V_2_ receptor on the blood-side membrane surface of collecting duct principal cells and interferes with the migration of water channels to the cell membrane, thus inhibiting water reabsorption. Recommendations based on the results of clinical use in Japan limit the loop diuretic dose to as low as 20 mg/day or up to 40 mg/day, refrain from increasing the dose, and add tolvaptan at an early stage if further dose increases are required. The combined use of tolvaptan with no increase in the furosemide dose at an early stage before severe deterioration in the estimated glomerular filtration rate (eGFR) or the occurrence of hyponatremia improves the prognosis of patients with hepatic cirrhosis.^5,6^ Tolvaptan may exert a very strong aquaretic effect, particularly at the beginning of its administration; therefore, the first dose requires hospitalization. Adequate hydration and careful observation are required when tolvaptan is administered to older adult patients with terminal cancer who are prone to dehydration. Particularly, in patients with terminal cancer, conventional eGFR values based on serum creatinine levels may overestimate renal function because such patients have low capacities of protein synthesis and creatinine production; therefore, caution should be exercised when initiating treatment with tolvaptan.^7^

Moreover, in a postmarketing surveillance of tolvaptan,^8^ many patients had received loop diuretics before the use of tolvaptan because the latter is indicated for use only in patients who do not adequately respond to other diuretics. Dohi et al. recommended the use of K-sparing diuretics before the initiation of tolvaptan treatment.^9^ They highlighted that prior use of furosemide may reduce the efficacy of tolvaptan because tolvaptan also uses the osmotic pressure difference between the interstitium and ureter to exert the diuretic effect. In accordance with this, at our hospital, we select diuretic agents other than loop diuretics for patients undergoing diuretic therapy for the first time.

The standard treatment for the management of edema in patients with terminal cancer is the comprehensive use of multiple procedures such as skin care, compression therapy, and manual lymph drainage. In addition, daily life guidance is important. To our knowledge, no effective drug therapy regimens for lymphatic edema are currently available. Surgical treatment includes lymphaticovenular anastomosis; however, its effectiveness has not been demonstrated particularly in patients with terminal cancer, and no established effective procedures are available for the treatment of edema in such patients. To date, we have administered tolvaptan to 29 patients diagnosed with refractory ascites/edema associated with hepatic cirrhosis at our hospital. The mean duration of administration was 18.5 days, which was shorter than that for the commonly used drugs because of a short survival period in our patients. Nevertheless, the treatment improved the activities of daily living in some patients with terminal cancer, such as recovery of walking ability because of the resolution of lower leg edema. PS scores at the initiation of tolvaptan treatment were ≥3 in >50% of patients, indicating that they were in the terminal stage, PS3 in 10, and PS4 in 8. However, all patients received tolvaptan orally because once-daily administration was sufficient. No patients experienced adverse events such as hypernatremia after the initiation of tolvaptan treatment, indicating that it could be used safely. The use of tolvaptan may be expanded to include patients with terminal cancer because of the ease and safety of administration.

Regarding the effects of tolvaptan, for the 20 patients who survived for ≥1 month and for whom we could confirm changes in their body weight, >50% showed a decrease in their body weight (12 patients; 60.0%). For 12 patients showing a decrease in weight, the nutritional status remained unchanged or improved, which improved ascites retention and edema and alleviated symptoms. However, body weight did not decrease, and ascites or edema did not improve in eight patients; therefore, the selection of patients expected to respond, after accumulation of data from more patients, is necessary in future studies. Moving forward, we should collect data from more patients to study the patient characteristics that determine responsiveness to tolvaptan treatment.

In conclusion, tolvaptan is safe for the treatment of fluid retention refractory to conventional diuretics in patients with terminal cancer who are able to receive it orally, and is effective in some patients. While we are awaiting data from more patients to determine the characteristics of patients responsive to tolvaptan, tolvaptan use may be expanded to include patients with terminal cancer.

## Figures and Tables

**Table1:** Characteristics of patients

Factor	n=29
Age (years, median)	72 (46–93)
Gender (male/female)	13/16
Body weight (kg, median)	56.9 (34.9–106.3)
Cancer type (cases)	
Gastorenterol	21
Lung	2
Gynaecological	2
Urology	2
Others	2
PS (1/2/3/4)	2/9/10/8
Fluid collection	
Whole+PE+AS	12
Leg edema only	8
PE and/or AS	8
Whole only	1
Hospital stay (median)	38 (9–345)

PS: performance status, PE: pleural effusion, AS: ascites

**Table2:** Laboratory data on admission

Factor	n=29
Albumin (g/dL)	2.3 (1.2–4.2)
Transthyretin (mg/dL)	8.9 (2.1–38.2)
BUN (mg/dL)	19.9 (8.6–49.3)
Serum creatinine (mg/dL)	0.81 (0.38–2.25)
Serum sodium (mEq/L)	136 (123–142)
Serum potassium (mEq/L)	4.2 (2.6–5.0)

## Presentation

We presented these results at the 25th Congress of Japan Society of Palliative Medicine.

## Supplementary Material

PDF-Japanese
