# Supplementary material for: Efficacy and safety of tolvaptan for refractory fluid collection and edema in the terminal cancer patients
Source: Fujita Med J. 2022 May 25;9(1):8–11. doi: 10.20407/fmj.2021-005 (PMC9923446; doi:10.20407/fmj.2021-005)
Supplement: Supplementary file 1 — PDF-Japanese [file fmj-9-008-s001.pdf]

序論

Tolvaptan は日本で開発された vasopressin V_2 受容体アンタゴニストであり，水のみを尿中に排泄する経口利尿薬である．入院での投与開始が必要とされるが、心不全や肝硬変での臨床経験の蓄積により tolvaptan の有効性や安全性は証明されてきている[1-3]．終末期がん患者においても著明な体液貯留をきたすことがあり，tolvaptan 以外の経口利尿薬が投与されるが，明らかな治療効果が得られないことも多い．この難治性体液貯留は薬剤抵抗性のことが多く，同時に浮腫が増悪しないように食事量や輸液量を制限することとなり，必要な栄養がとれなくなる場合もある．また著明な浮腫がとれず，腹水や胸水貯留により腹部膨満や呼吸困難を併発し ADL の低下をもたらし長期入院の原因となりとなることがある．心不全の利尿薬として開発された tolvaptan が肝硬変に伴う腹水貯留に有効とされ報告されているが，緩和医療領域では未だあまり使われていない利尿薬であり，肝転移などにより肝障害を来たし難治性腹水と低アルブミン血症を伴う終末期がん患者では，少なくとも代償性から非代償性への過渡期の肝硬変と診断できるため[4]，このような症例については積極的に tolvaptan の投与を行っている．

そこで今回難治性の著明な体液貯留をきたした終末期がん患者に対して tolvaptan 投与を行い，その投与効果と安全性について検討した．

方法

2017 年 8 月から 2020 年 2 月までに著明な体液貯留をきたし，他の利尿薬で効果の得られなかった終末期がん患者 29 名を対象として tolvaptan 7.5mg/day を経口投与した．前医からの継続処方は 7 名で，22 名に当院にて新規投与を行った．前投薬としては，ループ利尿薬が 19 例，K 保持性利尿薬 11 例，サイアザイド系利尿薬 1 例で投薬なしが 3 例で， 5 例が 2 剤を内服していた．対象の内訳は，男性 13 名，女性 16 名（46 歳から 93 歳、中央値 72 歳）で，原発巣は消化器 21 名（膵 5 名， 68 胆道 4 名，肝臓 3 名，食道 1 名，胃 3 名，結腸 3 名，直腸 2 名），肺 2 名，婦人科領域 2 名，泌尿器 2 名，その他２名であった（table 1）．体重および栄養評価として serum level of albumin (Alb)， serum level of transthyretin （TTR），腎機能として BUN，クレアチニン(Cr)および電解質の測定を行った．この測定は最低月に１回測定を行った．

本研究は，藤田医科大学医学研究倫理審査（受付番号 HM20-105：終末期がん患者における CRP/TTR 比，TTR，CRP の予後予測能の検討）のもとに後方視的に行った．

統計学的処理は IBM SPSS Statistics version 24.0 for Macintosh (International Business Machines Co., Armonk, NY, USA)を用いて行い，有意差検定に連続変数は Student's t-test あるいは Mann Whitney U test を用いた．

結果

Tolvaptan 投与期間は 1 日から 85 日（平均 18.5 日)であった．投与開始時の performance status(PS) は PS1 2 名，PS2 9 名，PS3 10 名，PS4 8 名であり，PS3 以上が 18 名 62.1% と PS 低下例が過半数を占めていた．しかし，全例で経口摂取可能であった．体液貯留は，体幹浮腫および胸腹水貯留が 12 例，下腿浮腫のみ 8 名，腹水貯留のみ 5 名，胸水貯留のみ 2 名，胸腹水貯留 1 名，全身浮腫のみ 1 名であった．生存期間は中央値 38 日(9-345)であった（Table 1)．

入院時の体重は中央値 56.9 kg(34.9-106.3)であった．投与 1 か月後の PS は，PS の評価可能であった 14 例において，PS1 1 名，PS2 5 名，PS3 4 名，PS4 4 名であり，PS3 以上が 8 名(57.1%)と投与開始時の PS とほぼ変わらなかった．PS の変化ついては改善 3 名，不変 8 名，悪化 3 名であり，終末期がん患者においても 11 名(78.6%)で PS は維持・改善された．1 か月以上の体重変化を確認できたのは 20 名で中央値 57.6kg （35.2-99.4）で，体重減少を半数以上の 12 名（60.0%）に認め，増加 8 名（40.0%）であった．体重減少した 12 名の減少量の中央値は 3.1 kg/ 月(0.8-7.5)で，順調に減っていた．一方，体重増加した 8 名の中央値は 1.7 kg/ 月(0.3-9.4) で， refractory cachexia に陥った 6.6kg 増と 9.4kg 増の 2 例を除くと， 5 例が 2kg 以内の増加であり，減少症例に比べて増加量は緩やかであった(Figure 1).

また下腿浮腫の 8 名中 6 名に改善を認めた.

栄養状態は，Alb は入院時全例で測定しており，その中央値は 2.3 g/dL(1.2-4.2)と著明に低く，28 例で測定し TTR では，その中央値も入院時 8.9 mg/dL(2.1- 38.2)と異常低値であった（Table 2)．

1 か月後の Alb は，測定できた 24 例の中央値で 2.3g/dL(0.8-2.9)と入院時と変わらず，1 か月以上生存できた方では若干の低下を認めたが有意差なく保たれていた（p=0.420, Figure 2)．

1 か月後の TTR について測定できた 21 例で，中央値は 8.6 mg/dL(0.8-23.7)で，減少量は 0.3 mg/dL であり，21.8mg/dL 減少した 1 例を除くと，1 か月以上生存できた患者ではほとんど変化なく保たれており，5 例に改善例も認めた（p=0.359, Figure 2)．2 か月後、3 か月後で測定できた症例は数例であり，統計的な結果は得られなかった.

腎機能については，BUN と Cr は入院時全例で測定して，BUN の中央値は 19.9 mg/dL(8.6-49.3)で，20mg/dL 以上は 12 例(41.4%)で，Cr では 0.81mg/dL(0.38-2.25)で 1.0 g/dL 以上は 9 例(31.0%)で，入院時すでに腎機能障害を持っている症例を 30%以上に認めた（Table 2)．1 か月後の BUN は、測定できた 22 例の中央値は 23.4 mg/dL(13.5-34.0)と入院時に比べて軽度の上昇に留まっていた(p=0.209, Figure 3)．1 か月後の Cr は測定できたのは 21 例で，その中央値は 0.91 mg/dL(0.39-2.41)でほとんど変化なく，特にもともと 1.0 未満の正常値であった症例では 1.0 を超える悪化例はなかった（p=0.869,

Figure 3）．こちらも 2 か月後、3 か月後で測定できた症例は数例であり，統計的な結果は得られなかった．

電解質については血清 Na が入院時の中央値 136 mEq/dL(123-142)と全例で高 Na 血症を認めず（Table 2），1 か月後の中央値 135 mEq/dL(124-142)でほとんど tolvaptan 導入による影響は認めなかった（p=0.532, Figure 4）．血清 K についても入院時の中央値 4.2 mEq/dL(2.9-5.0)で，全例において重篤な高 K 血症を認めず（Table 2），1 か月後の中央値 4.5 mEq /dL(3.2-4.5)と高カロリー輸液を行ったことと低 K を是正したため上昇したが，正常化したのであり，tolvaptan 導入による影響は認めず，透析導入例や不整脈等の副作用も認めなかった（p=0.041, Figure 4）．

考察

最近，選択的競合的 vasopressin 受容体拮抗薬である tolvaptan の登場により，腹水を有する肝硬変患者における利尿剤治療の推奨レジメンが変わってきた．Tolvaptan は腎集合管主細胞の血液側細胞膜にある vasopressin V_2 受容体を選択的に拮抗し、水チャネルの細胞膜への移動を抑制することで水の再吸収を阻害する．本邦における多施設での使用経験において，ループ利尿薬の投与量は 20mg/day もしくは 40mg/day 程度までの少量投与の段階にとどめておき，それ以上の増量の必要性が生じた際には早期に tolvaptan を併用することが推奨されている．高度の eGFR 低下や低ナトリウム血症が生じない早期の段階にてフロセミドの増量をせず tolvaptan を併用することで肝硬変の予後改善にもつながると考えられている [5,6]．Tolvaptan は特に投与開始時には水利尿効果が非常に強く現れることもあり，初回投与は入院で投与することになっている．十分な水分補給を行い，脱水に陥りやすい高齢者で，しかも終末期がん患者に対する投与は特に注意深く観察する必要がある．特に終末期がん患者では，蛋白合成が低く，クレアチニンの産生が落ちているため低い値を呈することが多く，血清クレアチニンを使った一般的な eGFR では腎機能を過大評価する可能性があり導入には注意が必要である [7]．また，tolvaptan の市販後調査が行われ [8]，tolvaptan の使用適応が他の利尿薬による効果がない場合に限られるためであり，前投薬としてループ利尿薬が多く使われていた．

一方, 土肥らは tolvaptan 投与の前利尿薬として K 保持性利尿薬の投与を推奨している [9]. これは､tolvaptan の利尿効果は, 間質と尿管の間の浸透圧の違いも利用しているため, tolvaptan の効果がフロセミドの前投与によって低下してしまう可能性があることを指摘している．このため当科でも新規で使用する場合はループ以外の利尿薬の投与を行っている．

終末期がん患者の浮腫の原因は様々であり，その標準治療はスキンケア，圧迫療法，用手的リンパドレナージなどを包括的に行うことであり，日常生活指導も重要とされているが，現時点では，終末期がん患者の浮腫に対する効果的な薬物療法はない．リンパ浮腫に対する手術療法として，リンパ管細静脈吻合術などが行われているが，終末期がん患者の

浮腫に対して，その有効性は示されておらず，終末期がん患者における浮腫の有効な治療法は確立されていない．

これまで当院において肝硬変に伴う難治性胸腹水や浮腫と診断した 29 名に tolvaptan を投与した．投与期間は平均 18.5 日で生存期間が短いため一般的に使われている使用期間より短いが，下腿浮腫がとれたために歩行可能となったなど終末期の ADL 向上に寄与した症例も認められた．投与開始時 PS は PS3 10 名、PS4 8 名と半数以上が 3 以上と終末期の状態であったが，1 日 1 回の内服で良いことから全例経口摂取可能であった．また全例で tolvaptan 投与による高 Na 血症などの有害事象は認めず，安全に使用することができた．飲みやすく安全に投与可能であることから，今後終末期のがん患者においても適応拡大していくことが期待される．

本研究において，1 か月以上生存し，体重変化を確認できた 20 名中，体重減少は半数以上の 12 名（60.0%）に認められ，これらの 12 名で栄養状態は横ばいから改善し，胸腹水貯留や浮腫の軽減を認めたものの，8 名では体重減少を認めず，胸腹水や浮腫も改善しなかった．しかしながら，1 か月後の PS が確認できた 14 名において，PS の改善および不変が 11 名（78.6%）であり，対象患者が終末期がん患者であることを考慮すると，この薬剤の有効性を示すものと考えられる．

今後症例の蓄積により，効果の期待できる症例の検討が必要と考えられる．

結論

Tolvaptan 投与は，終末期がん患者において一般的な利尿薬ではコントロール困難であった難治性体液貯留に対しても，経口摂取可能な患者に対して安全に投与可能で有効例を認めた．ただし，どのような症例において tolvaptan 投与が有効であるかは今後症例の蓄積が待たれるが，終末期がん患者に対する適応拡大が期待される．

この論文の要旨は第 25 回日本緩和医療学会で発表した．
著者に COI はありません．

引用文献

1. Felker GM, Mentz RJ, Cole RT, Adams KF, Egnaczyk GF, Fiuzat M, Patel CB, Echols M, Khouri MG, Tauras JM, Gupta D, Monds P, Roberts R, O'Connor CM. Efficacy and Safety of Tolvaptan in Patients Hospitalized With Acute Heart Failure. J Am Coll Cardiol. 2017; 69: 1399-406

2. Hiramine Y, Uojima H, Nakanishi H, Hiramatsu A, Iwamoto T, Kimura M, Kawaratani H, Trai S, Yoshiji H, Uto H, Sakaida I, Izumi N, Okita K, Koike K. Response

criteria of tolvaptan for the treatment of hepatic edema. J Gastroenterol. 2018; 53: 258–68
3. Sakaida I, Kawazoe S, Kajimura K, Saito T, Okuse C, Takaguchi K, Okada M, Okita K, ASCITES-DOUBLEBLIND Study Group. Tolvaptan for improvement of hepatic edema: A phase 3, multicenter, randomized, double-blind, placebo-controlled trial. Hepatol Res. 2014; 44: 73–82
4. Fukui H, Saito H, Ueno Y, et al. Evidence-based clinical practice 187 guidelines for liver cirrhosis 2015. J Gastroenterol. 2016; 51: 629-50
5. Nagayama I, Masuda T, Nakagawa S, Murakami T, Ohara K, Matsuoka R, Kobayashi T, Maeshima A, Akimoto T, Saito O, Muto S, Nagata D. Different Effects on Fluid Distribution between Tolvaptan and Furosemide in a Liver Cirrhosis Patient with Chronic Kidney Disease. Intern Med. 2019; 58: 1587-91
6. Sakaida I, Terai S, Kurosaki M, Okada M, Hirano T, Fukuda Y. Real-world effectiveness and safety of tolvaptan in liver cirrhosis patients with 195 hepatic edema: results from a post-marketing surveillance study (START study). J Gastroenterol. 2020; 55:800-10
7. Uekuzu Y, Higashiguchi T, Futamura A, Chihara T, Usui M. Influence of muscle mass on the estimation of glomerular filtration rate in Japanese terminal cancer patients. Clin Exp Nephrol. 2020; 24: 876-84
8. Iwamoto T, Maeda M, Saeki I, Hidaka I, Tajima K, Ishikawa T, Takami T, Sakaida I. Analysis of tolvaptan non-responders and outcomes of tolvaptan treatment of ascites. J Gastroenterol Hepatol. 2019; 34: 1231-5
9. Dohi K, Watanabe K, Ito M. Urine Osmolality-Guided Tolvaptan Therapy in Decompensated Heart Failure. Circ J. 2013; 77: 313-4
